# Carbuncle due to *Salmonella* Enteritidis: a novel presentation

**DOI:** 10.1186/s13099-017-0200-2

**Published:** 2017-09-11

**Authors:** Wadha Alfouzan, Dieter Bulach, Hidemasa Izumiya, Khaled AlBassam, Simin Sheikh, Nasser Alrubai’aan, M. John Albert

**Affiliations:** 1 Department of Microbiology, Faculty of Medicine, Kuwait University, Jabriya, Kuwait; 2 Microbiological Diagnostic Unit Public Health Laboratory, Peter Doherty Institute for Infection and Immunity and Melbourne Bioinformatics, The University of Melbourne, Victoria, Australia; 3 National Institute of Infectious Diseases, Tokyo, Japan; 4 Department of Surgery, Farwaniya Hospital, Al Farwaniyah, Kuwait; 5 Microbiology Unit, Farwaniya Hospital, Al Farwaniyah, Kuwait

**Keywords:** *S.* Enteritidis, Carbuncle, Whole genome sequencing, Virulence genes

## Abstract

**Background:**

*Salmonella* Enteritidis causes intestinal and extra-intestinal infections, but rarely cutaneous infections. It has never been reported to cause carbuncle (a collection of interconnected furuncles with multiple pustular openings). We report a case of carbuncle due to *S. *Enteritidis.

**Case presentation:**

An adult Bangladeshi patient with type 2 diabetes presented with a carbuncle on the left-side of his neck. A pure culture of *S.* Enteritidis was grown from the pus of the carbuncle. The patient was successfully treated with ciprofloxacin to which the isolate was susceptible. Whole genome sequencing of the strain showed that it possessed three additional virulence genes—*pef* (for plasmid-encoded fimbriae), *spv* (for salmonella plasmid virulence), *rck* (for resistance to complement killing) -responsible for systemic infections that were absent in the genome of a reference *S.* Enteritidis strain. In phylogenetic analysis, the strain clustered with other *S.* Enteritidis strains from different parts of the world.

**Conclusions:**

A weakened immune system of the patient due to diabetes mellitus and the additional virulence genes of the isolate may have contributed to the unusual presentation of carbuncle. The possibility of *S. *Enteritidis to cause carbuncle should be considered.

**Electronic supplementary material:**

The online version of this article (doi:10.1186/s13099-017-0200-2) contains supplementary material, which is available to authorized users.

## Background

Non-typhoid *Salmonella* infections lead to not only self-limited acute gastrointestinal infections, but also, bacteremia with or without extra-intestinal focal infections [1, 2]. Extra-intestinal focal infections include septic arthritis, osteomyelitis, cholangitis, aortitis, endocarditis, pneumonia, urinary tract infection, and meningitis [1, 2]. Non-typhoid salmonellae do not produce cutaneous infections in immunocompetent individuals, but a single case of leg abscesses has been reported in an immunocompromised patient [3]. Here we report carbuncle in a patient with type 2 diabetes mellitus. Carbuncle is an aggregate of connected furuncles (hair follicle infections) with multiple pustular openings. To our knowledge, this is the first case of carbuncle due to non-typhoid salmonellosis. Clinical manifestation is the result of interaction between the host and the pathogen. In this report, we also investigated whether there are unique genetic properties of the *Salmonella* isolate that could have contributed to this unusual clinical manifestation. This was achieved by whole genome sequencing of the isolate.

## Case presentation

A 55-year-old Bangladeshi male with a history of type 2 diabetes mellitus and hypertension presented to the Emergency Department of Farwaniya Hospital, Kuwait in December 2015 with a left-sided neck swelling (8 cm × 6 cm) discharging pus through multiple sinuses. He was suffering from this condition for the past 2 weeks. On examination on alert, voice, pain and unresponsive (AVPU) scale, he was conscious and oriented with stable signs and without fever (oral temperature of 36.7 °C). Examinations covering the respiratory, cardiovascular, gastrointestinal and respiratory systems were unremarkable. His body mass index (BMI) was 27.4 (classified as overweight [4] and a risk factor for type 2 diabetes [5]). Blood examination by flow cytometry (Sysmex 9000, Bornbarch, Norderstedt, Germany) showed leukocytosis (18 × 10^9^/L, mainly neutrophils, normal range is 3.7 × 10^9^/L)) and by oxygen rate method (Beckman Coulter DxC 800, Brea, CA, USA) hyperglycemia (random blood sugar of 20 mmol/L; normal range is 3.9–6.1 mmol/L). A diagnosis of carbuncle with hyperglycemia was made. He was given parenteral insulin on a sliding scale (1–14 units with increments of 1–2 units during a 24 h period). Incision and drainage of the carbuncle were done and pus was sent for microbiological studies. An empirical therapy with clindamycin (300 mg intravenously every 8 h) was started suspecting infection with *Staphylococcus aureus* which is a common cause of carbuncle [6]. This therapy was continued for 3 days until culture and susceptibility report became available (see below).

**Fig. 1:**
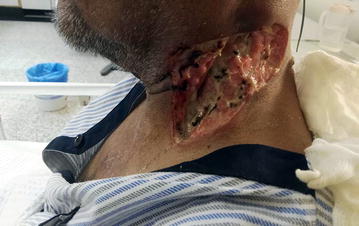
Photo of debrided carbuncle on the left-side of patient’s neck after successful antibiotic therapy

Pus was cultured on blood agar, MacConkey agar, chocolate agar and gentamicin blood agar. Blood agar and MacConkey agar were incubated aerobically, chocolate agar microaerobically and gentamicin blood agar anaerobically. Incubation was done at 37 °C for 24–48 h. All plates except the gentamicin plate grew a pure culture (organism was susceptible to gentamicin, see below) which was identified as a *Salmonella* species using Phoenix method (Becton–Dickinson, Franklin Lakes, NJ, USA) and Vitek 2 and Vitek-MS methods (Biomerieux, Marcy l’Etoile, France). Blood, urine and stool were sent for culture. Blood culture was done using BD BACTEC system (Becton–Dickinson) and there was no growth of any organism. Urine culture was done on blood agar and cysteine-, lactose-, and electrolyte-deficient (CLED) agar. There was no bacteriuria and *Salmonella* species was not isolated. Stool was cultured on MacConkey agar, Campy agar and *Salmonella*–*Shigella* agar (SSA) and enriched in selenite F broth with subsequent subculture on SSA. No bacterial diarrheal pathogen including *Salmonella* species was isolated. In vitro susceptibility of the *Salmonella* isolated from carbuncle (designated as CSE76F) to antibiotics was done by Vitek II system (bioMerieux) and E test (AB Biodisk, Solna, Sweden) and interpreted by Clinical and Laboratory Standards Institute (CLSI) guidelines [7]. It was susceptible to amikacin, amoxicillin–clavulanic acid, chloramphenicol, tetracycline, ceftazidime, ceftriaxone, cefuroxime, cephalothin, imipenem, meropenem, ciprofloxacin, gentamicin, piperacillin–tazobactam, tigecycline, and trimethoprim–sulfamethoxazole, but resistant to ampicillin and clindamycin. The antibiotic therapy was changed to ciprofloxacin (400 mg intravenously every 12 h) for 14 days at which time he recovered. This prolonged intravenous therapy was necessitated to avoid relapse because the patient was immunocompromised (due to diabetes mellitus) and the wound was large. After debridement of the wound (Fig. 1), the patient was referred to plastic surgery for skin grafting.

**Fig. 2:**
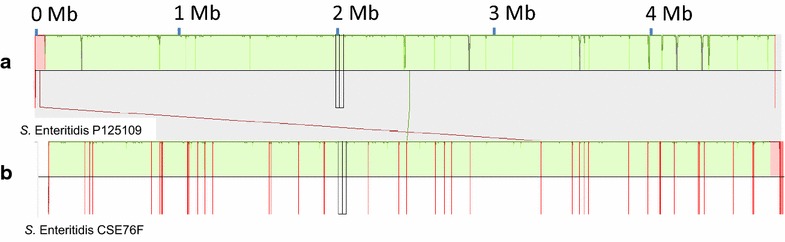
A graphical overview of comparison of genome sequence of reference strain P125109 (**a**) with draft genome sequence of strain CSE76F (**b**). No evidence of insertions/deletions or genomic rearrangements was seen in CSE76F. The figure shows 61 contigs in CSE76F aligned to the P125109 genome. Vertical red lines in B show the edges of contigs

**Fig. 3:**
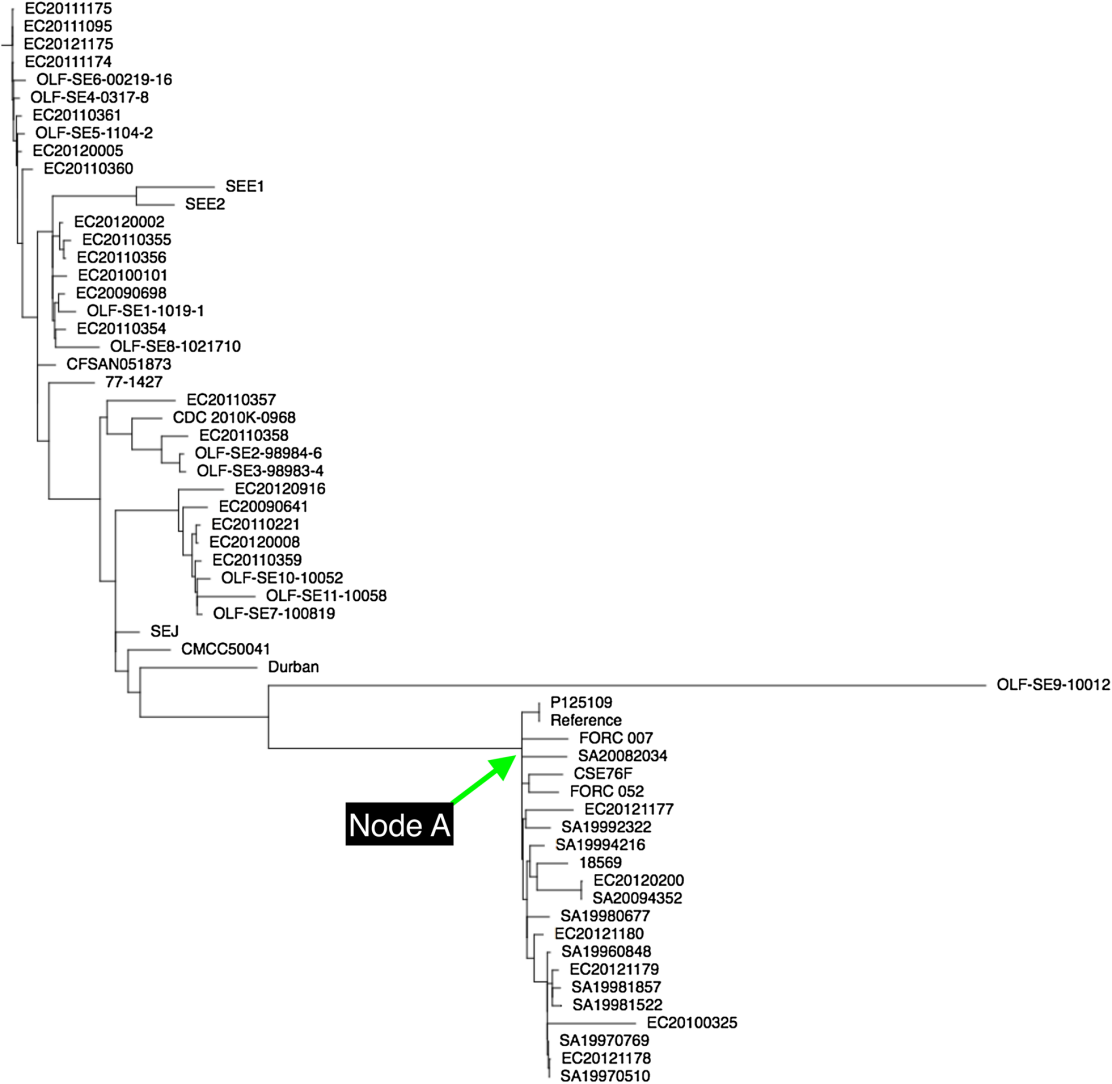
Core genome comparison shows a cluster of strains with core genome sequences that are less than 100 SNPs different from each other (indicated as “Node A”). Strain P125109 was used as the reference genome sequence for the core genome comparison. All pairwise genome comparisons covered more than 97% of the reference genome sequence. Among the 60 sequences compared in the tree, there were 2452 aligned positions for which there was a sequence difference in one or more of the genome sequences of the strains

The *Salmonella* organism was typed as Enteritidis species by multilocus sequence typing (MLST) [8] and a *sefA* (*S* *almonella* Enteritidis fimbria)*A gen*e-specific PCR [9]. A draft genome sequence for isolate CSE76F was determined using the whole genome shotgun method. The sequencing library was prepared using the Nextera XT DNA sample preparation kit (Illumina, San Diego, CA) and the sequence read data were produced on the Illumina NextSeq instrument (paired end, 150 base reads). A total of 3,213,288 reads yielded 477,626,349 bases of usable sequence data after filtering to remove low quality sequence data and adapter sequences (approximately 100- fold read coverage of the genome) (Sequence Read Archive [SRA] Accession Number, SRR5198927). De novo assembly of the read data with MegaHit [10] yielded a draft genome sequence comprising a total of 4,743,613 bases in 61 contigs (minimum contig size 500 bases). The MLST type was confirmed from the draft genome sequence (Senterica scheme, ST 11) from the genome sequence [11] and the antimicrobial resistance gene profile of the isolate was determined using Abricate [12] and the ResFinder database [13]. This showed that the isolate carried a *bla*_TEM-1b_ gene (encoding a class A beta-lactamase). This fits with the observed resistance of the isolate to ampicillin [14]. The most related closed genome sequence was that from *Salmonella enterica* subsp. enterica serovar Enteritidis strain P125109 isolated from an outbreak of human food-poisoning (RefSeq: NC_011294) with 55 SNPs identified across 98.82% of the strain P125109 genome sequence. Aligning the CSE76F contigs to the P125109 genome sequence revealed no apparent major genomic deletions, inversions or rearrangements (Fig. 2). Analysis of the virulence gene profile was performed using Abricate with the VFDB database of virulence genes [15]. CSE76F strain possessed three additional virulence genes-*pef* (*p* *lasmid*-*e* *ncoded* *f* *imbriae* with all four subunits-*pefA*, *pefB, pefC, pefD*), *spv* (*s* *almonella* *p* *lasmid* *v* *irulence* with all three subunits- *spvA, spvB, spvC*) and *rck (* *r* *esistance to* *c* *omplement* *k* *illing*)-that were absent in strain P125109. Phylogenetic relationship of CSE76F strain based on single nucleotide polymorphism (SNP) with that of 60 closed genomes of *S.* Enteritidis strains available in GenBank (shown in Additional file 1: Table S1) was constructed using FastTree [16]. Core SNP differences were called using Nullarbor [17]. Strain CSE76F did not occupy a unique position, but clustered with several other strains from the United Kingdom, South Korea, Canada and the United States of America (Fig. 3).

## Discussion and conclusions

*pef* gene mediates adhesion and biofilm formation [18], *spv* gene cytotoxicity [19] and *rck* gene serum-resistance and survival inside the macrophage [20]. All the three genes are located in a 60 MDa virulence plasmid that enhances the growth rate during the systemic phase of the disease [21]. It is not surprising that the strain CSE76F possessed the additional virulence genes which would have contributed to its ability to produce the unusual manifestation of carbuncle in the immunologically weakened diabetes mellitus patient. However, the strain clustered with several other *S.* Enteritidis strains from different parts of the world.

Since there was no history of diarrhea in this patient or in his close family contacts, he would have had an asymptomatic intestinal infection with *S. *Enteritidis. The organism would have entered the blood stream from the intestinal tract and then the skin. Another possibility that the patient might have introduced the organism into his skin by scratching it by fecally contaminated fingers. This case shows that the possibility of non-typhoid *Salmonella* (*S.* Enteritidis) causing carbuncle should be taken into consideration.

## Additional file

**Additional file 1: Table S1.** Complete genome sequences to which strain CSE76F was compared.

## Abbreviations

AVPU: alert voice pain unresponsive scale
CLED: cysteine lysine electrolyte deficient agar
SSA: *Salmonella-Shigella* agar
CLSI: Clinical and laboratory Standards Institute
*sef*: *Salmonella* Enteritidis fimbria*A*
*pef*: plasmid-encided fimbriae
*spv*: *Salmonella* plasmid virulence
*rck*: resistance to complement killing
SRA: equenced read archive
SNPs: single nucleotide polymorphisms
BMI: body mass index
